# Prevalence and factors associated with fear of childbirth in late pregnancy: a cross-sectional study

**DOI:** 10.3389/fpubh.2025.1589568

**Published:** 2025-05-30

**Authors:** Dongning He, Jinling Zhang, Guoyu Wang, Yan Huang, Na Li, Mingxin Zhu, Yangxue Meng, Biru Luo

**Affiliations:** ^1^Department of Nursing, West China Second University Hospital, Sichuan University/ West China School of Nursing, Sichuan University, Chengdu, China; ^2^Key Laboratory of Birth Defects and Related Diseases of Women and Children (Sichuan University), Ministry of Education, Chengdu, China; ^3^Department of Obstetric Nursing, West China Second University Hospital, Sichuan University/ West China School of Nursing, Sichuan University, Chengdu, China

**Keywords:** fear of childbirth, mental health, maternal health, prenatal care, late pregnancy

## Abstract

**Background:**

Fear of childbirth is a common concern among pregnant women, potentially affecting maternal physical and mental health as well as birth outcomes. However, the factors influencing fear of childbirth are still being explored. This study aims to assess the prevalence of fear of childbirth among women in late pregnancy and identify associated factors, with a particular focus on pregnancy comorbidities, complications, and specific obstetric conditions.

**Methods:**

A cross-sectional study was conducted from October 2022 to March 2024 at a tertiary women’s and children’s hospital in western China. A total of 6,335 pregnant women were recruited. The Childbirth Attitude Questionnaire was used to measure fear of childbirth. Data on pregnancy comorbidities, complications, and specific obstetric conditions were extracted from the hospital information system. Ordinal logistic regression was performed to analyze factors associated with different levels of fear of childbirth.

**Results:**

The mean score of the Childbirth Attitude Questionnaire was 34.69 ± 8.15. 79.8% of the participants reported mild to severe fear of childbirth in late pregnancy. Ordinal logistic regression analysis identified several significant risk factors for severe fear of childbirth, including lower education level, primiparity, no history of uterine scarring, natural conception, intention to have a cesarean section, high myopia, and urinary disease.

**Conclusion:**

Fear of childbirth is highly prevalent among women in late pregnancy and is influenced by multiple factors, including pregnancy comorbidities and complications. Obstetric healthcare providers should be aware of fear of childbirth and consider both sociodemographic and obstetric characteristics when implementing targeted interventions and counseling to support high-risk groups.

## 1 Introduction

Pregnancy and childbirth are significant stages in a woman’s life. During this period, women transition into motherhood while experiencing significant physiological and psychological changes. In addition to routine prenatal care for pregnant women, the identification and timely management of psychological issues should not be neglected. Fear of childbirth (FOC) is a psychological condition characterized by anxiety or unease during pregnancy and birth, which can significantly impact the physical and psychological well-being of pregnant women during pregnancy and childbirth (1), even in the postpartum period (2). Epidemiological data show that the prevalence of FOC varies widely, ranging from 12 to 58.6% across different countries and regions (2, 3), which is influenced by cultural factors, healthcare standards, and the tools used to measure FOC (4). Despite varying rates, studies consistently highlight the widespread prevalence and severity of FOC. Approximately 33% of pregnant women experience FOC in late pregnancy, with 11–14% experiencing severe fear and requiring medical intervention (2).

Research shows that FOC in pregnant women changes as pregnancy progresses (5, 6), and the prevalence of FOC significantly increases in late pregnancy (7). FOC in late pregnancy may have varying degrees of short-term and long-term effects on maternal and neonatal outcomes. In the short term, FOC influences pregnant women’s preferences for birth methods, the progression of birth, and the childbirth experience. Evidence suggests that FOC is a significant factor contributing to the increase in unnecessary cesarean sections (8, 9) because of the declining willingness of women to undergo vaginal birth (10). Severe FOC may lower the pain threshold, heighten pain perception, and magnify the sensation of labor pain (11, 12), leading many women to select cesarean sections as a safer and less painful choice. In terms of the progression of birth, FOC can result in increased plasma catecholamine levels, which may cause insufficient uterine contractions and prolong the stages of birth (13, 14). An extended duration of birth often increases discomfort, further reinforcing pregnant women’s demand for medical interventions, including cesarean sections and epidural analgesia (15, 16). FOC can significantly impact maternal and neonatal outcomes, as well as future pregnancies. If women experience severe FOC during their current pregnancy, that experience can result in adverse fetal outcomes, including abnormal heart rhythms, low APGAR scores, and low birth weight (14). Additionally, women may experience post-traumatic stress disorder after birth, which could recur in subsequent pregnancies (17). Women who experience FOC are more likely to develop psychological disorders in the postpartum period, such as postpartum depression and anxiety, which may further impact their willingness and breastfeeding behavior (18). The severity of FOC varies between individuals, and the duration of its long-term effects also differs. Therefore, identifying factors influencing FOC in late pregnancy, providing accurate childbirth information, and implementing targeted interventions are crucial for decreasing the impact of FOC.

Previous research on factors influencing FOC primarily focuses on sociodemographic or psychological variables, such as maternal age, educational level, anxiety, or depression (19, 20), while the impact of pregnancy comorbidities, complications, or specific obstetric conditions remains underexplored. Therefore, we conducted a large-scale cross-sectional study to (1) investigate the prevalence of FOC in late pregnancy and (2) identify its associated factors, particularly pregnancy comorbidities, complications, and specific obstetric conditions, which may aid obstetric healthcare providers in accurately identifying and managing high-risk individuals with severe FOC.

## 2 Methods

### 2.1 Setting

The study was conducted from October 2022 to March 2024 at a women’s and children’s tertiary hospital in Sichuan Province, China. As a leading referral center in western China, the hospital receives a high volume of deliveries each year and provides care for pregnant and postpartum women from a wide catchment area, including both urban and rural regions across multiple provinces. It specializes in the management of high-risk and critically ill pregnancies, offering services for a broad range of obstetric conditions and diverse demographic groups. This setting provides a valuable foundation for investigating maternal health issues, including fear of childbirth, in regional and under-resourced populations. Pregnant women who undergo routine prenatal checks at this hospital have a unique identification number, which helps distinguish individuals by their names and allows healthcare staff to identify them accurately.

### 2.2 Participants

This study used a consecutive sampling strategy, enrolling all eligible pregnant women who met the inclusion criteria and provided informed consent. The inclusion criteria were as follows (1): Age ≥18 years (2); Gestational age ≥28 weeks (3); Participants with singleton pregnancies (4); Absence of mental illness; and (5) Participants were required to communicate effectively in Mandarin and complete the questionnaires independently. In this study, “primiparous” refers to women who have not previously given birth to a viable infant, which may include those who have had prior pregnancies but no deliveries. This is distinct from “first-time pregnant” women, who are experiencing their first pregnancy. As the study design involved the full inclusion of all eligible participants during the data collection period, no formal *a priori* sample size calculation was conducted. However, the final sample of 6,335 participants provided a sufficiently large and representative dataset for robust multivariable analysis.

### 2.3 Measurement

Sociodemographic and selected obstetric information were collected through a self-designed questionnaire. Sociodemographic information included age, ethnicity, residence, education level, employment status, and monthly per capita family income. Selected obstetric information included gestational age, number of pregnancies, whether conceived by *in vitro* fertilization-embryo transfer (IVF-ET), and intended mode of childbirth. Additional obstetric information, such as pregnancy comorbidities, complications, and specific obstetric conditions, was obtained from the hospital information system by matching each participant’s identification number. Participants’ FOC was assessed with the Childbirth Attitude Questionnaire (CAQ). This instrument was originally designed by Areskog et al. (21), further developed by Lowe (22), and Tanglakmankhong (23). It was subsequently translated and validated for use in Chinese populations by Juan et al. (24). The translation process followed standard procedures, including forward and backward translation, expert review, and pilot testing. The CAQ contains 16 items and is scored using a 4-point Likert scale from 1 (not afraid) to 4 (very afraid). Scores ranging from 16 to 27, 28 to 39, 40 to 51, and 52 to 64 represent low, mild, moderate, and severe FOC, respectively. A high score indicates that a woman is fearful. The Chinese version of the CAQ has been widely used in studies involving Chinese pregnant women and has demonstrated good applicability and measurement properties in this context (25, 26). In the present study, the scale also showed excellent internal consistency, with a Cronbach’s alpha coefficient of 0.902.

### 2.4 Data collection

We used paper questionnaires for data collection. Four investigators, trained by the principal researchers, recruited pregnant women in late pregnancy during routine antenatal checks at the obstetric clinic to complete the questionnaires. Before participation, all participants were required to sign an informed consent form and offer their correct identification number, then they could receive the questionnaires. To protect participant privacy, the identification number was used solely for matching questionnaire data with the Hospital Information System and was not linked to names, phone numbers, or other personally identifiable information. No identifiable data were collected on the paper questionnaires. Only authorized members of the research team had access to the ID-hospital data mapping file, which was securely stored in a password-protected system. Participants had the right to decline participation or withdraw from the study at any time. Investigators reviewed the questionnaires on the spot. If any items were left blank, participants were prompted to fill them in immediately. However, questionnaires with more than 20% missing data were considered invalid and excluded. Following data collection, two investigators independently cross-checked the questionnaires to ensure accuracy. Subsequently, the main researcher and another researcher entered the data into the Epidata software. Pregnancy comorbidities, complications, and obstetric information were retrieved from the Hospital Information System using the participants’ identification number. These clinical diagnoses were recorded in the Hospital Information System by attending physicians. The diagnoses followed the International Classification of Diseases, 10th Revision (ICD-10) coding system and were based on established national clinical guidelines. The hospital employs a standardized multi-level quality control process for medical records to ensure diagnostic accuracy and consistency, including physician verification, departmental audits, and periodic reviews by the medical records department. To enhance analytical reliability, we categorized clinically related conditions into broader diagnostic groups as described in Table 1, which helped address potential variations in specific coding practices. Finally, data from the paper questionnaires were matched with the corresponding hospital records. We received a total of 6,335 valid questionnaires.

**Table 1:** Pregnancy comorbidities, complications, and specific obstetric conditions (*N* = 6,335).

Variable	*N*	%
Pregestational or gestational diabetes mellitus	1,115	17.6
Hypertensive disorder complicating pregnancy	108	1.7
Pregnancy with heart disease	650	10.3
High myopia	130	2.1
Thyroid disease	1,106	17.5
Breast disease	218	3.4
Hepatobiliary diseases	376	5.9
Urinary disease	139	2.2
Adnexal diseases	1,126	17.8
Hematological disease	349	5.5
Immune system disease	284	4.5
Infectious disease	279	4.4
High risk of thrombosis	224	3.5
Fetal abnormalities	707	11.2
Placental or umbilical cord abnormalities	384	6.1
Abnormal amniotic fluid volume	288	4.5

### 2.5 Data statistics

All statistical analyses were performed using IBM SPSS 27.0 software. The total score of the Childbirth Attitude Questionnaire, as a continuous variable, was normally distributed and presented as mean ± standard deviation (SD). Sociodemographic, obstetric information, and levels of FOC were categorical variables, shown as frequencies (n) and percentages (%). As the categorical variables did not follow normal distributions, non-parametric tests were used to identify potential factors influencing levels of FOC. The Mann–Whitney U test was used for two-group comparisons, and the Kruskal-Wallis H test was applied for comparisons among multiple groups. To further explore factors independently associated with different levels of FOC, variables that were significantly associated with FOC in the univariate analysis (*p* < 0.05) were simultaneously entered into an ordinal logistic regression model. Adjusted odds ratios (ORs) and 95% confidence intervals (CIs) were calculated to assess the strength of association while controlling for potential confounding among predictors. The proportional odds assumption was tested using the test of parallel lines in SPSS. The results showed that this assumption was not violated (*p* > 0.05), indicating that the ordinal logistic regression model was appropriate for the data. A two-tailed *p*-value of less than 0.05 was considered statistically significant.

### 2.6 Ethics approval

This study was approved by the Ethics Committee of West China Second University Hospital, Sichuan University, with ethics approval number 2022 (191). Informed consent was obtained from all participants. Participants identified as having a severe FOC based on questionnaire responses were not directly intervened with by the research team. However, their results were flagged and communicated to their designated obstetricians during antenatal care. A corresponding note was placed in their medical records to prompt closer emotional observation. In cases where obstetricians observed signs of significant psychological distress (e.g., anxiety or depression), a referral to the hospital’s psychological counseling clinic was advised, following standard clinical pathways.

## 3 Result

### 3.1 Sociodemographic and obstetric characteristics of participants

A total of 14.0% of participants were of advanced maternal age (≥35 years). Approximately 57.7% (n = 3,658) of the participants were at or beyond 37 weeks of gestation. A total of 65.3% of participants were pregnant for the first time, while 93.8% were primiparous. Additionally, 192 participants had a history of adverse pregnancy outcomes. 11.9% (n = 756) of participants conceived through IVF-ET. Furthermore, 67.8% (n = 4,297) of the participants intended to have a vaginal birth. The specific demographic and obstetric characteristics are presented in Table 2.

**Table 2:** Sociodemographic and obstetric characteristics of participants (*N* = 6,335).

Variables	*N*	%
Age, yr		
<25	77	1.2
25~	2,013	31.8
30~	3,360	53.0
≥35	885	14.0
Ethnicity		
Han	6,150	97.1
Others	185	2.9
Residence		
Urban	5,578	88.0
County/Town	663	10.5
Rural	94	1.5
Education Level		
High school and below	191	3.0
Junior college	1,028	16.2
Undergraduate	3,518	55.6
Master’s degree and above	1,598	25.2
Employment Status		
Employed	5,105	80.6
Not employed	1,230	19.4
Family per capita monthly income, CNY		
<3,000	79	1.2
3,000~	481	7.6
5,000~	2,407	38.0
>10,000	3,368	53.2
Gestational age, wk		
<37	2,677	42.3
≥37	3,658	57.7
Number of pregnancies		
Once	4,137	65.3
Twice	1,440	22.7
Thrice and above	758	12.0
Primiparous		
Yes	5,940	93.8
No	395	6.2
Adverse pregnancy history		
Yes	192	3.0
No	6,143	97.0
A history of uterine scarring		
Yes	150	2.4
No	6,185	97.6
Other abdominal surgery history		
Yes	191	3.0
No	6,144	97.0
In vitro fertilization-embryo transfer		
Yes	756	11.9
No	5,579	88.1
Intended mode of birth		
Vaginal birth	4,297	67.8
Cesarean section	2,038	32.2

### 3.2 Pregnancy comorbidities, complications, and specific obstetric conditions of participants

We retrieved all participants’ pregnancy comorbidities, complications, and related abnormalities from the hospital information system and categorized these conditions as shown in Table 1. Thyroid diseases in the classification included hypothyroidism, hyperthyroidism, Hashimoto’s thyroiditis, and thyroid cancer. Breast diseases comprised hyperplasia of mammary glands, breast cysts, and breast cancer. Hepatobiliary diseases included hepatic cysts, liver hemangiomas, hepatitis, intrahepatic cholestasis of pregnancy, cholecystolithiasis, gallbladder polyps, and cholecystitis. Urinary diseases included hydronephrosis, renal cysts, nephrolithiasis, and ureteral calculi. Adnexal diseases encompassed cervical cancer, uterine fibroids, adenomyosis, ovarian cysts, and polycystic ovary syndrome. Hematological diseases included iron deficiency anemia, Mediterranean anemia, and thrombocytopenia. Immune system diseases included antiphospholipid syndrome and systemic lupus erythematosus. Infectious diseases included viral hepatitis and syphilis. Pregestational or gestational diabetes mellitus (17.6%), thyroid disease (17.5%), and adnexal disease (17.8%) were the most prevalent comorbidities. Hypertensive disorders complicating pregnancy (1.7%), high myopia (2.1%), and urinary disease (2.2%) had the lowest prevalence.

Among other special obstetric conditions, fetal abnormalities were the most prevalent, accounting for 11.2%. These mainly included fetal growth restriction, congenital heart disease, renal collecting system separation, or abnormalities in the appearance. Placental and umbilical cord abnormalities included velamentous cord insertion, placenta previa, and single umbilical artery. Amniotic fluid abnormalities included polyhydramnios and oligohydramnios.

### 3.3 Participants’ levels of fear of childbirth

The mean total score of the questionnaires was 34.69 ± 8.15. Based on these scores, 79.8% (n = 5,060) of the pregnant women experienced varying degrees of FOC during late pregnancy. Specifically, 53.5% (n = 3,391) of the participants reported mild FOC, 1,466 (23.1%) reported moderate fear, and 203 (3.2%) reported severe FOC.

### 3.4 Individual participant characteristics and fear of childbirth

Table 3 compares the levels of FOC among participants with different characteristics. Older pregnant women (≥35 years) reported a higher proportion of no FOC (24.9%) compared to younger groups (*p* = 0.002). The level of FOC differed significantly among participants with different educational levels (p = 0.002). First-time pregnant women reported higher levels of fear than women who had two or more pregnancies (*p* = 0.001). Primiparous women reported higher levels of FOC compared to multiparous women (*p* < 0.001). Women with a history of uterine scarring showed lower levels of FOC compared to those without (p < 0.001). Women who conceived via IVF-ET had lower levels of fear compared with women who had natural pregnancies (p < 0.001). A statistically significant difference (p < 0.001) was observed in the level of FOC between participants intending to have a vaginal birth and those intending to have a cesarean section. Statistically significant differences in FOC were also observed among participants with certain complications. These included high myopia (*p* = 0.025), thyroid disease (*p* = 0.044), urinary disease (*p* = 0.038), and hematological disease (p = 0.044).

**Table 3:** Comparison of fear of childbirth of participants differing by characteristics (*N* = 6,335).

Variable	No *n* (%)	Mild *n* (%)	Moderate n (%)	Severe *n* (%)	*p-*value
Age, yr					0.002
<25	16(20.8)	34 (44.1)	21 (27.3)	6 (7.8)	
25~	382 (19.0)	1,064 (52.8)	499 (24.8)	68 (3.4)	
30~	657 (19.5)	1,835 (54.6)	762 (22.7)	106 (3.2)	
≥35	220 (24.9)	458 (51.7)	184 (20.8)	23 (2.6)	
Ethnic Groups					0.462
Han	1,244 (20.2)	3,283 (53.4)	1,424 (23.2)	199 (3.2)	
Others	31 (16.7)	108 (58.4)	42 (22.7)	4 (2.2)	
Residence					0.362
Urban	1,112 (19.9)	3,013 (54.0)	1,272 (22.8)	181 (3.3)	
County/Town	144 (21.7)	334 (50.4)	166 (25.1)	19 (2.9)	
Rural	19 (20.2)	44 (46.8)	28 (29.8)	3 (3.2)	
Education Level					0.002
High school and below	57 (29.8)	83 (43.5)	44 (23.0)	7 (3.7)	
Junior college	200 (19.4)	519 (50.5)	269 (26.2)	40 (3.9)	
Undergraduate	700 (19.9)	1,905 (54.2)	796 (22.6)	117 (3.3)	
Master’s degree and above	318 (19.9)	884 (55.3)	357 (22.3)	39 (2.5)	
Employment Status					0.074
Employed	996 (19.5)	2,750 (53.9)	1,198 (23.5)	161 (3.1)	
Not employed	279 (22.7)	641 (52.1)	268 (21.8)	42 (3.4)	
Family per capita monthly income, CNY					0.090
<3,000	17 (21.5)	33 (41.8)	27 (34.2)	2 (2.5)	
3,000~	99 (20.6)	249 (51.8)	115 (23.9)	18 (3.7)	
5,000~	463 (19.2)	1,344 (55.8)	533 (22.2)	67 (2.8)	
>10,000	696 (20.7)	1,765 (52.4)	791 (23.5)	116 (3.4)	
Gestational age, wk					0.624
<37	523 (19.5)	1,430 (53.4)	634 (23.7)	90 (3.4)	
≥37	752 (20.6)	1,961 (53.6)	832 (22.7)	113 (3.1)	
Number of pregnancies					0.001
Once	769 (18.6)	2,240 (54.1)	992 (24.0)	136 (3.3)	
Twice	318 (22.1)	772 (53.6)	307 (21.3)	43 (3.0)	
Thrice and above	188 (24.8)	379 (50.0)	167 (22.0)	24 (3.2)	
Primiparous					<0.001
Yes	1,149 (19.3)	3,189 (53.7)	1,405 (23.7)	197 (3.3)	
No	126 (31.9)	202 (51.1)	61 (15.5)	6 (1.5)	
Adverse pregnancy history					0.963
Yes	40 (20.8)	102 (53.1)	45 (23.5)	5 (2.6)	
No	1,235 (20.1)	3,289 (53.6)	1,421 (23.1)	198 (3.2)	
A history of uterine scarring					<0.001
Yes	49 (32.7)	75 (50.0)	24 (16.0)	2 (1.3)	
No	1,226 (19.8)	3,316 (53.6)	1,442 (23.3)	201 (3.3)	
Other abdominal surgery history					0.110
Yes	50 (26.2)	90 (47.1)	47 (24.6)	4 (2.1)	
No	1,225 (19.9)	3,301 (53.7)	1,419 (23.1)	199 (3.3)	
In vitro fertilization-embryo transfer					<0.001
Yes	201 (26.6)	408 (54.0)	129 (17.0)	18 (2.4)	
No	1,074 (19.2)	2,983 (53.5)	1,337 (24.0)	185 (3.3)	
Intended mode of birth					<0.001
Vaginal birth	924 (21.5)	2,346 (54.6)	917 (21.3)	110 (2.6)	
Caesarean section	351 (17.2)	1,045 (51.3)	549 (26.9)	93 (4.6)	
Pregestational or gestational diabetes mellitus					0.735
Yes	233 (20.9)	586 (52.5)	256 (23.0)	40 (3.6)	
No	1,042 (20.0)	2,805 (53.7)	1,210 (23.2)	163 (3.1)	
Hypertensive disorder complicating pregnancy					0.942
Yes	22 (20.4)	55 (50.9)	27 (25.0)	4 (3.7)	
No	1,253 (20.1)	3,336 (53.6)	1,439 (23.1)	199 (3.2)	
Pregnancy with heart disease					0.373
Yes	121 (18.6)	360 (55.4)	143 (22.0)	26 (4.0)	
No	1,154 (20.3)	3,031 (53.3)	1,323 (23.3)	177 (3.1)	
Pregnancy with high myopia					0.025
Yes	19 (14.6)	62 (47.7)	43 (33.1)	6 (4.6)	
No	1,256 (20.2)	3,329 (53.7)	1,423 (22.9)	197 (3.2)	
Pregnancy with thyroid disease					0.044
Yes	220 (19.9)	559 (50.5)	291 (26.3)	36 (3.3)	
No	1,055 (20.2)	2,832 (54.1)	1,175 (22.5)	167 (3.2)	
Pregnancy with breast disease					0.190
Yes	38 (17.4)	120 (55.1)	48 (22.0)	12 (5.5)	
No	1,237 (20.2)	3,271 (53.5)	1,418 (23.2)	191 (3.1)	
Pregnancy with hepatobiliary diseases					0.542
Yes	70 (18.6)	196 (52.1)	95 (25.3)	15 (4.0)	
No	1,205 (20.2)	3,195 (53.6)	1,371 (23.0)	188 (3.2)	
Pregnancy with urinary disease					0.038
Yes	19 (13.7)	74 (53.2)	37 (26.6)	9 (6.5)	
No	1,256 (20.3)	3,317 (53.5)	1,429 (23.1)	194 (3.1)	
Pregnancy with adnexal diseases					0.125
Yes	253 (22.5)	587 (52.1)	246 (21.8)	40 (3.5)	
No	1,022 (19.6)	2,804 (53.9)	1,220 (23.4)	163 (3.1)	
Pregnancy with hematological disease					0.044
Yes	67 (19.2)	188 (53.9)	74 (21.2)	20 (5.7)	
No	1,208 (20.2)	3,203 (53.5)	1,392 (23.2)	183 (3.1)	
Pregnancy with immune system disease					0.501
Yes	48 (16.9)	162 (57.1)	64 (22.5)	10 (3.5)	
No	1,227 (20.3)	3,229 (53.3)	1,402 (23.2)	193 (3.2)	
Pregnancy with infectious disease					0.089
Yes	58 (20.8)	140 (50.2)	65 (23.3)	16 (5.7)	
No	1,217 (20.1)	3,251 (53.7)	1,401 (23.1)	187 (3.1)	
High risk of thrombosis					0.968
Yes	46 (20.5)	119 (53.1)	53 (23.7)	6 (2.7)	
No	1,229 (20.1)	3,272 (53.6)	1,413 (23.1)	197 (3.2)	
Fetal abnormalities					0.164
Yes	157 (22.2)	385 (54.5)	148 (20.9)	17 (2.4)	
No	1,118 (19.9)	3,006 (53.4)	1,318 (23.4)	186 (3.3)	
Placental or umbilical cord abnormalities					0.836
Yes	77 (20.1)	212 (55.2)	85 (22.1)	10 (2.6)	
No	1,198 (20.1)	3,179 (53.4)	1,381 (23.2)	193 (3.1)	
Abnormal amniotic fluid volume					0.491
Yes	66 (22.9)	142 (49.3)	70 (24.3)	10 (3.5)	
No	1,209 (20.0)	3,249 (53.7)	1,396 (23.1)	193 (3.2)	

### 3.5 Factors influencing the level of fear of childbirth in late pregnancy

The aforesaid statistically significant variables were used as inputs for ordinal logistic regression analysis (Table 4). Age, number of pregnancies, pregnancy with thyroid disease, and hematological disease were not statistically significant in the regression equation (*p* > 0.05). Participants with a junior college education had significantly higher levels of FOC than those with a master’s degree and above (OR = 1.221, 95%CI: 1.047, 1.424), while no significant difference was observed in other education levels. Primiparous women had a high risk of severe FOC levels compared to multiparous women (OR = 0.627, 95%CI: 0.498, 0.788). Women without a history of uterine scarring had higher levels of FOC compared to those with a history of uterine scarring (OR = 1.588, 95%CI: 1.132, 2.229). Women who conceived naturally had higher levels of FOC compared to those who conceived via IVF-ET (OR = 1.636, 95%CI: 1.404, 1.905). Participants intending to have a cesarean section had higher levels of FOC compared to those intending a vaginal birth (OR = 1.510, 95%CI: 1.361, 1.675). Pregnant women without high myopia (OR = 0.711, 95%CI: 0.511, 0.990) and urinary disease (OR = 0.726, 95%CI: 0.527, 0.999) had a lower level of FOC compared to those with these conditions.

**Table 4:** Logistic regression analysis of factors influencing the level of fear of childbirth.

Variable	β	SE	Wald	*p*	OR (95%CI)
Age, yr. (Ref: ≥35)					
<25	0.292	0.230	1.611	0.204	1.339 (0.853, 2.103)
25~	0.126	0.083	2.302	0.129	1.134 (0.964, 1.334)
30~	0.099	0.075	1.721	0.190	1.104 (0.952, 1.279)
Education Level (Ref: Master’s degree and above)					
High school and below	−0.080	0.150	0.280	0.597	0.924 (0.688, 1.240)
Junior college	0.200	0.078	6.479	0.011	1.221 (1.047, 1.424)
Undergraduate	0.057	0.058	0.943	0.332	1.058 (0.944, 1.186)
Number of pregnancies (Ref: Thrice and above)					
Once	0.045	0.083	0.292	0.589	1.046 (0.888, 1.232)
Twice	−0.055	0.088	0.381	0.537	0.947 (0.796, 1.126)
Primiparous (Ref: Yes)					
No	−0.467	0.117	15.963	<0.001	0.627 (0.498, 0.788)
A history of uterine scarring (Ref: Yes)					
No	0.463	0.173	7.171	0.007	1.588 (1.132,2.229)
In vitro fertilization-embryo transfer (Ref: Yes)					
No	0.492	0.078	40.026	<0.001	1.636 (1.404, 1.905)
Intended mode of birth (Ref: Vaginal birth)					
Caesarean section	0.412	0.053	60.504	<0.001	1.510 (1.361, 1.675)
Pregnancy with high myopia (Ref: Yes)					
No	−0.341	0.169	4.071	0.044	0.711 (0.511, 0.990)
Pregnancy with thyroid disease (Ref: Yes)					
No	−0.119	0.063	3.530	0.060	0.888 (0.784, 1.005)
Pregnancy with urinary disease (Ref: Yes)					
No	−0.321	0.163	3.864	0.049	0.726 (0.527, 0.999)
Pregnancy with hematological disease (Ref: Yes)					
No	−0.051	0.105	0.239	0.625	0.950 (0.773, 1.168)

## 4 Discussion

### 4.1 The fear of childbirth prevalence among women in late pregnancy

In this cross-sectional study, we investigated the prevalence and influencing factors of FOC among 6,335 women in late pregnancy. We focused on pregnancy comorbidities, complications, and specific obstetric conditions. We found that 1,669 participants (26.3%) experienced moderate to severe FOC during late pregnancy. The mean FOC score was 34.69 (SD = 8.15), which was higher than those reported in similar studies using the same tool in other regions of China (25–27). This difference might be due to differences in gestational age or previous childbirth experience, which significantly affect levels of FOC (25, 28, 29). The score in our study was higher than that reported in Greece (30), but lower than that of women in Pakistan (31). It is important to recognize that countries may differ significantly in living conditions, ethnicity, religion, and social structures, all of which may influence the perception and assessment of the FOC (27). Additionally, questionnaire scores may not fully capture the complexity of FOC. Consequently, we further analyzed the prevalence of FOC and found that 79.8% of women in late pregnancy experienced varying levels of fear. This percentage was calculated by combining the proportions of participants categorized as having mild, moderate, or severe FOC based on the CAQ scoring criteria. The result is similar to a study conducted in Chongqing, also a city in western China, in which the reported prevalence of 75.15% was measured with the same assessment tool (32). However, it was higher than the level reported in a coastal city in eastern China (20). This variation may be associated with differences in healthcare quality, socioeconomic factors, and access to healthcare between eastern and western China; healthcare quality is known to affect FOC (33). Additionally, our study setting as a tertiary referral center in western China may have contributed to the higher prevalence, as our population included more high-risk pregnancies and patients from diverse socioeconomic backgrounds and underserved areas. Moreover, the prevalence of FOC in our study was slightly higher than that reported in Middle Eastern countries, such as Egypt and Iran, where prevalence ranged from 70.4 to 71.5% (14, 34). These differences could be attributed to differences in religious and cultural norms (4). The fact that prevalence exceeds 70% regardless of geographic, cultural, or social conditions emphasizes the fact that FOC is a common issue among women in late pregnancy.

In our study, 3.2% of participants experienced severe FOC, a proportion comparable to that reported in a study from Finland (35), but lower than the 8.1% reported in Turkey and 12% in Brazil (36, 37). These differences may be related to the use of different measurement tools, but they also reflect the widespread prevalence and potential clinical implications of FOC. It is important for obstetric healthcare providers to be attentive to the FOC, particularly among women with severe FOC, and to implement targeted interventions.

### 4.2 Influencing factors of fear of childbirth in late pregnancy

On the basis of demographic factors, we identified numerous variations in FOC among women in late pregnancy, and we examined the presence of pregnancy comorbidities, complications, and special obstetric conditions. Our findings show that young women are more likely to experience moderate to severe FOC. These findings are similar to research by Elsharkawy et al., and they suggest that young women may be more prone to high levels of fear because of their lack of experience and uncertainty in the childbirth process (34). Our results contradict reports of Huang et al. and Räisänen et al., who found that older pregnant women had higher levels of fear, possibly because of concerns about their own health or potential pregnancy complications (25, 28). However, our regression analysis revealed that age was not a statistically significant factor influencing the level of FOC in late pregnancy (*p* > 0.05); thus, factors other than age may be more influential. Nevertheless, women of all ages experience FOC. Therefore, it is essential to ensure that pregnant women of all age groups receive adequate attention and support to alleviate their fears. We found that women with a junior college education, compared with women having a master’s degree and above, were more likely to experience a higher level of FOC (OR = 1.221, 95%CI: 1.047, 1.424). An association between low educational level and severe fear in late pregnancy is consistent with the findings of Gao et al. and Laursen et al. (38, 39). Such an association may exist because women with low education lack knowledge about childbirth or have limited access to comprehensive health information (40), making them more prone to anxiety or worry. In contrast, women with high education levels tend to experience less fear, possibly because they easily obtain and understand information related to childbirth (41), which enhances their confidence. Thus, there may be a need to provide better support and health information to women who have more limited education (42).

First-time pregnant women, particularly primiparas, had a high prevalence of moderate to severe fear. The regression analysis demonstrated that being a primipara was statistically significant (*p* < 0.001), establishing it as an important factor influencing the level of FOC in late pregnancy. This finding is similar to research by Adams et al., and it suggests that primiparas have a higher FOC because of their lack of practical childbirth experience and uncertainty about the birth process (13), particularly labor pain (43). These results revealed the need for obstetric healthcare professionals to provide enhanced support and education to primiparas, instructing them in acquiring knowledge about childbirth. We found that pregnant women with a history of uterine scarring experienced less FOC in late pregnancy, and this condition was a significant influence in regression analyses (*p* = 0.007, OR = 1.588, 95%CI: 1.132, 2.229). A scarred uterus is usually the result of a cesarean section or other uterine surgery. Women who have experienced such surgery may have a greater awareness of the risks of birth; thus, they have more realistic expectations of subsequent births. However, our finding contrasts with the findings of Sluijs and Hou (44, 45). Phunyammalee et al. found that the level of childbirth fear of women with a history of uterine scarring due to previous cesarean sections was similar to that of women without cesarean sections (46). This finding may be attributed to the fact that experienced women received better care during their previous cesarean sections, which resulted in reduced fear of subsequent childbirth. Although there are some differences in results, they all suggested that healthcare providers need to ensure that appropriate support and attention is provided to this group. Our results showed that women with natural pregnancies had higher fear levels than women who conceived via IVF-ET, and planned vaginal childbirth was a significant factor influencing FOC in late pregnancy. This finding may be due to the fact that women who conceive via IVF-ET usually receive more medical monitoring and psychological support because of the longer treatment process, which could enhance their understanding of pregnancy and birth. Women with natural pregnancies may have lacked this intensive medical support, leading to more anxiety about birth. Women intending to have cesarean section had higher fear levels than women planning vaginal births (OR = 1.510, 95%CI = 1.361, 1.675), which is similar to findings by Størksen and Haines (47, 48). The preference of pregnant women for cesarean section may exist because of a strong fear of labor pain (49), the prolonged duration of birth (50), and the belief that natural birth is more unpredictable (51). Additionally, the intended mode of birth is a significant factor in the FOC, suggesting that understanding a woman’s birth intention and providing targeted psychological support and information about birth options may help to alleviate the FOC.

Pregnant women with high myopia, thyroid disease, urinary disease, or hematological disease had higher levels of FOC in late pregnancy, with high myopia and urinary disease identified as influencing factors of FOC in this study. Our results show that pregnant women without myopia had 0.711 times (95%CI = 0.511, 0.990) lower risk of severe FOC in late pregnancy compared with women who had myopia. Some pregnant women fear that exertion during vaginal birth could lead to retinal detachment, but there is no evidence that high myopia increases the risk of retinal detachment during birth (52). Among our study participants, urinary disease was mainly nephritis or hydronephrosis. Birth may increase the burden on the kidneys (53), which may be a plausible explanation for why pregnant women with urinary disease fear that childbirth could harm their urinary health. Although thyroid disease and hematological diseases were not identified as factors influencing FOC, they are still worth considering. Pregnant women with thyroid disease may need to continue medication during pregnancy; fluctuations in thyroid hormone levels can affect emotions, leading to anxiety or depression (54), which may increase FOC. Furthermore, some pregnant women had anemia, which could raise concerns about the risk of blood loss during vaginal birth or cesarean section, thereby raising FOC. Although these conditions do not directly influence FOC, they may indirectly increase anxiety and fear related to childbirth. Therefore, based on the analysis of these pregnancy comorbidities, complications, and specific obstetric conditions, our findings suggest that certain maternal health conditions may be associated with a higher risk of severe FOC. This may provide a basis for identifying potentially high-risk groups in late pregnancy. These findings do not serve as direct intervention recommendations, but rather highlight theoretical risk patterns that may assist clinicians in identifying patients who warrant additional attention during antenatal care. Further prospective and interventional studies are needed to determine whether such risk-based approaches can be translated into effective support strategies.

### 4.3 Limitation

There are several limitations to this study. First, the single-center design and regional setting in a tertiary referral hospital in western China may limit the generalizability of the findings. The study population included a high proportion of high-risk pregnancies and women in late gestation, which could contribute to the relatively high prevalence of FOC. In addition, factors such as family support, prior counseling, and cultural differences were not examined and may further influence the patterns of FOC. Second, the study sample was predominantly primiparous (93.8%), and the number of participants with certain comorbidities, such as high myopia or urinary disease, was relatively small. Although we statistically adjusted for parity and other covariates, the sample structure may limit generalization to more diverse obstetric populations. Further studies with more balanced and diagnosis-specific samples are warranted. Third, we focused on the independent effects of each variable and did not assess potential interaction effects among predictors. Additionally, the use of self-reported measures may introduce subjective bias. Future studies should explore the combined effects of risk factors and consider integrating qualitative methods for a more comprehensive assessment of FOC.

## 5 Conclusion

Fear of childbirth is highly prevalent among women in late pregnancy and is influenced by a combination of demographic, obstetric, and clinical factors. In this large cross-sectional study, primiparity, lower education levels, natural conception, and a preference for cesarean delivery were all significantly associated with higher levels of FOC. Additionally, specific comorbidities and complications, such as high myopia and urinary diseases, were identified as independent predictors of severe FOC. These findings highlight the importance of integrating FOC screening into routine prenatal care, particularly among high-risk groups. By recognizing the multidimensional nature of FOC, obstetric healthcare providers can offer more personalized counseling and support strategies to alleviate maternal fear, promote informed birth choices, and potentially improve maternal psychological well-being and birth outcomes.

## Data Availability

The original contributions presented in the study are included in the article/supplementary material, further inquiries can be directed to the corresponding authors.
